# A newly identified left–right asymmetry in larval sea urchins

**DOI:** 10.1098/rsos.160139

**Published:** 2016-08-31

**Authors:** Jason Hodin, Keegan Lutek, Andreas Heyland

**Affiliations:** 1 Friday Harbor Laboratories, University of Washington, Friday Harbor, WA, USA; 2 Department of Integrative Biology, University of Guelph, Guelph, Ontario, Canada

**Keywords:** development, plasticity, fluctuating asymmetry, morphometrics, larval ecology, evolution

## Abstract

Directional asymmetry (DA) in body form is a widespread phenomenon in animals and plants alike, and a functional understanding of such asymmetries can offer insights into the ways in which ecology and development interface to drive evolution. Echinoids (sea urchins, sand dollars and their kin) with planktotrophic development have a bilaterally symmetrical feeding pluteus larva that undergoes a dramatic metamorphosis into a pentameral juvenile that enters the benthos at settlement. The earliest stage of this transformation involves a DA: a left-side invagination in mid-stage larvae leads to the formation of the oral field of the juvenile via a directionally asymmetric structure called the echinus rudiment. Here, we show for the first time in two echinoid species that there is a corresponding DA in the overall shape of the larva: late-stage plutei have consistently shorter arms specifically on the rudiment (left) side. We then demonstrate a mechanistic connection between the rudiment and arm length asymmetries by examining rare, anomalous purple urchin larvae that have rudiments on both the left and the right side. Our data suggest that this asymmetry is probably a broadly shared feature characterizing ontogeny in the class Echinoidea. We propose several functional hypotheses—including developmental constraints and water column stability—to account for this newly identified asymmetry.

## 1. Background

Many species of benthic invertebrates have a planktonic larval phase, which may allow these taxa to exploit alternative resources across life-history stages, increase their dispersal ability and maintain connectivity among populations [1–3]. The echinoderms—including sea urchins, sea stars and sea cucumbers—exhibit a wide variety of such planktonic larval forms, both feeding and non-feeding [4–8]. These forms are the result of evolutionary pressures that appear to shape larval morphology within the confines of opposing functional constraints, in particular on feeding ability versus stability in the water column [9,10]. Specifically, feeding structures generally require large surface area for particle capture, whereas stability, especially in turbulent waters, relies upon minimal surface area [8].

Consistent with these proposed trade-offs are the derived, non-feeding larval forms that have evolved independently and repeatedly across echinoderm taxa. Such larvae avoid the aforementioned functional constraints on feeding versus stability, and thus tend to be relatively simple in overall structure, with uniform ciliation or multiple ciliated bands circling their spheroid bodies to facilitate movement [11]. By contrast, feeding larvae exhibit more complex morphologies, and two classes of echinoderms—the ophiuroids (brittle stars and basket stars) and the echinoids (sea urchins, sand dollars and kin)—have independently evolved similar-looking pluteus larvae [12,13], with 2–8 or more larval arms supported by internal skeletal rods. These arms are used for feeding and swimming, provide structural support and might assist in passively orienting the larvae and offering protection from predation [14–17].

The diverse echinoderm larvae described above share one key developmental feature: at a certain point in larval development, a directional asymmetry (DA) appears when juvenile structures begin to form internally on the left side in the otherwise bilaterally symmetrical larva. In most echinoids with feeding larvae, this asymmetry is first visible as an invagination of ectoderm on the left side that contacts a coelomic pouch, and they jointly transform into the *echinus rudiment* (or ‘rudiment’ for short), which ultimately forms the oral portion of the pentamerally symmetrical juvenile [18–22]. The juvenile structures will continue to grow and differentiate within the larva until it reaches metamorphic competence, at which point if it subsequently encounters suitable substrate, the larva will settle irreversibly on the benthos. The relationship between the juvenile and the larva is in a sense parasitic as the juvenile structures develop at the expense of larval growth (reviewed in [23]).

The directionally asymmetrical rudiment invagination is particularly well studied in the purple sea urchin, *Strongylocentrotus purpuratus*. Aihara & Amemiya [21] provided strong experimental evidence that the right side of the larva is largely responsible for differentiating the L–R axis: laterally bisected larvae (before rudiment invagination) all regenerate and develop to competence, but the larvae developing from the left halves rarely exhibited normal L–R patterning, whereas those from the right side almost always developed normally. More targeted removals of portions of the right side also resulted in larvae with abnormal L–R patterning.

Recent molecular evidence has further supported this scenario of right side control of the L–R asymmetry. The identified genes that appear to regulate L–R asymmetry in urchins encode two secreted growth factor-like proteins—Nodal and Lefty—and the Pitx2 paired-class homeodomain protein, all three of which are expressed primarily on the right side of the larva; their proper expression restricts rudiment formation to the left side [24]. Bone morphogenetic protein (BMP) signalling is then asymmetrically activated and is required for the development of left-sided structures and marker genes [25]. Additionally, an H,K-ATPase-like protein also appears to be important, most likely via either H^+^ or K^+^ gradients that occur upstream of the asymmetric gene expression of *nodal, lefty* and *pitx2* [26].

Here, we show that concomitant with this L–R asymmetry in rudiment formation is a consistent asymmetry of the larval arms in advanced echinoid pluteus larvae, the extent of which has not previously been described. We first document the asymmetry in two disparate echinoids—the sand dollar *Dendraster excentricus* and the purple sea urchin *Str. purpuratus*—separated by 250 million years, suggesting that this asymmetry may be a common feature among echinoids with feeding larvae. We further explore the phenomenon in *Str. purpuratus* to evaluate the possible connection between the rudiment and larval arm asymmetries, and by examining anomalous larvae with rudiments on both the left and right sides. We discuss our results in the context of several hypotheses concerning the function of this newly identified asymmetry in sea urchin plutei. In so doing, we highlight the ways in which directional asymmetries offer a unique window into how ecology and development work together to drive organismal evolution.

## 2. Material and methods

### 2.1. Source populations, maintenance of adults and larval cultures

For the characterization of the arm-length asymmetries in *D. excentricus* (Eschscholtz) sand dollar larvae, we used adults collected at low tide (−0.25 m) from a large, intertidal population in East Sound (Orcas Island, WA, USA) on 17 July 2015. The adults were maintained at Friday Harbor Labs (FHL; Friday Harbor, WA, USA) in flowing seawater in sand bins until spawning. On 26 August 2015, we spawned several adults by intracoelomic injection with 0.5 M KCl. We set up crosses from two females: one by standard methods [27] using sperm collected dry that same day from a single spawning male, the second by aspirating off the eggs from the aboral surface of a second female who began spawning after we returned her to an aquarium. This second female's eggs were already fertilized, therefore we are unsure of the paternity in this second cross (several males were also spawning in the aquarium at that time, including the male from the first cross). Thus, the two crosses were either half sibs from different mothers, non-sibs, or a mixture, and we maintained them separately throughout to ensure that any results we obtained could not be explained by the larvae having been derived from an aberrant female. We conducted fertilizations and all subsequent rearing steps in 0.45 µm millipore-filtered natural seawater (MFSW) at room temperature, which varied between 19 and 22°C.

Sand dollar embryos at this temperature hatch during the first day of development. Approximately 24 h after fertilization, we set up one jar from each cross at approximately 1 larva ml^−1^ of MFSW, and fed them a combination of *Rhodomonas spp.* (2.5 cells µl^−1^) and *Dunaliella tertiolecta* (3 cells µl^−1^), and kept them gently stirred using a gyratory shaker table. We changed their water every 2 days by reverse filtration of more than 95% of the water volume and gave the larvae fresh MFSW and food. On day 3 (comparable to soft tissue stage iii from [22]), we reduced the larval density to 0.2 larvae ml^−1^ MFSW, and maintained them at that density until day 9 (more or less equivalent to skeletogenic stage 10 from [22]) when we conducted all larval arm measurements.

For the characterization of the corresponding arm-length asymmetries in purple urchins as well as the ontogenetic characterizations and feeding trials, we used adult *Str. purpuratus* (Stimpson), collected at Slip Point (Clallam Bay, WA, USA) and maintained in subtidal cages suspended off the floating docks at FHL, fed throughout the year ad libitum with drift kelp (mainly blades of *Nereocystis leutkeana*). We spawned two males and two females on 27 March 2015 at FHL, by intracoelomic injection with 0.5 M KCl. We then set up the four pairwise fertilizations in MFSW using standard methods [27] at 11°C. We transported embryos the next day to the University of Washington (Seattle, WA, USA) and continued to maintain the cultures at 11°C. On day 5, when the embryos had reached the late prism/early 4-arm larval stage, we set up a single gallon jar in MFSW at approximately 1 larva ml^−1^ from equal proportions of the four fertilizations, fed them a combination of *D. tertiolecta* and *Rhodomonas spp.* as described above, and over about an hour, warmed the culture to 15°C in a shaking water bath, where we maintained all cultures for the remainder of the experiment. Every 2 days, we cleaned the cultures and fed them as described above.

On day 15, most of the larvae had reached the rudiment invagination stage (soft tissue stage i from [22]), at which point we reduced the larval density to approximately 0.75 larvae ml^−1^ MFSW, and then to approximately 0.5 larvae ml^−1^ MFSW on day 17, with food at the same density as previously. On day 20 (approx. soft tissue stage iv from [22]), we individually selected 1000 of the optimally developing larvae, only rejecting those (less than 20% of the larvae) that appeared significantly smaller than the average larva. In so doing, we reduced the density to 0.17 larvae ml^−1^, and fed them as before. This stepwise reduction in density was an attempt to limit bouts of larval cloning, which can be induced by sudden shifts in density (unpublished data), and would be expected to increase variability in arm length within cultures [28].

On day 25 (approx. skeletogenic stage 1 from [22]), we selected out 83 larvae into each of six jars with 500 ml MFSW (so still at 1 larva 6 ml^−1^) and randomly assigned each jar to one of two treatments: three replicate jars of high food (full ration of *Dunaliella *: *Rhodomonas* at 1 : 4 cells µl^−1^) and three replicate jars of low food (25% ration at 0.25 : 1 cells µl^−1^) for the remainder of the experiment. Approximately 50% of the larvae in the high food treatment had reached metamorphic competence by day 42; the low food larvae had not quite reached metamorphic competence by the time we concluded the experiment on day 45.

For the ‘double-rudiment’ experiment, we used purple urchins originally obtained from The Cultured Abalone Ltd. (Goleta, CA, USA) and that we have maintained at the Hagen Aqualab at the University of Guelph (Guelph, Ontario) in an artificial seawater system on an 8 L : 16 D photoperiod at 12°C and 34 ppt salinity, fed ad libitum with rehydrated *Kombu* kelp (*Laminaria spp*.), repeatedly spawning the same individuals as they become gravid again. Over the last several years (2011–2013), we repeatedly noted an unusually high proportion (approx. 2–5%; data not shown) of offspring of particular urchins from our Guelph colony that exhibited rudiments on both their right and the left sides—so-called ‘double rudiments’—a seeming hypertrophy of the small right side invagination typical of development in at least some echinoids (including the two species we examined here; see [29]). Note that, since the death of the particular adults from which we obtained offspring with enhanced double-rudiment occurrence, we have no longer observed this phenomenon in our Guelph colony, despite having made no notable changes to our water or culturing system. Therefore, we conclude that the double-rudiment-enriched larval cohort that we examined here derived from a specific maternal, paternal or genetic effect, and that we typically (and since 2014, exclusively) have seen the phenomenon only at the expected rate of fewer than 1% of larvae in a colony ([30]; J.H., K.L. and A.H., unpublished data).

In September 2013, we obtained gametes from one male and one female adult *Str. purpuratus* by intracoelomic injection following protocols described above, whose larvae later exhibited the enhanced double-rudiment phenotype. After fertilization and hatching, we set up cultures at an approximate density of 0.5 larvae ml^−1^ of 0.45 µm millipore-filtered artificial seawater (MFASW), agitated to prevent the larvae from settling out of the water column. We transferred larvae three times per week to clean beakers with new water and fed them with either *D. tertiolecta* or *Rhodomonas spp.* at 12 cells µl^−1^ or 6 cells µl^−1^, respectively. In the third week of development, we noticed that this larval cohort exhibited the enhanced double-rudiment phenotype. At 21 days postfertilization (PF), we measured nine stage-matched (see below) single- and double-rudiment sibling larvae, and assessed asymmetry as in the previous experiment.

### 2.2. Staging and measurements

In FHL, we measured ten live 9-day-old sand dollar larvae from each of the two crosses, gently immobilized on slides under raised cover glass, using an Olympus BH-2 microscope. In Seattle, we staged and measured live purple urchin larvae, immobilized as above, using a Leitz Wetzlar Ortholux microscope. For the purple urchins, we employed the staging scheme as outlined in [22]. Note that we used stage bins (as defined in the legend to figure 3) for the analyses of the urchin data in an attempt to equalize the numbers of individuals within each bin for this dataset. In both FHL and Seattle, we measured skeletal rod lengths on haphazardly chosen larvae using a calibrated ocular micrometer, and calculated the *z*-axis offset of each measured skeletal rod using the gradated focus knob, which we had calibrated using a slide and cover glass of known thickness (measured with a micron caliper).

To account for possible measurement error and/or bias in our measurements, we used larvae fertilized and reared in Guelph as described above, but from a 3 August 2015 fertilization. On day 21, we packed and shipped approximately 200 of these live larvae to Seattle overnight, to conduct the error measurements on the Leitz microscope set-up used for the majority of our data (see above). The larvae arrived in good condition on day 22 at approximately 13°C, and we conducted the error measurements on that day as follows. We haphazardly chose 20 larvae and placed them on individual microscope slides with raised cover glass as described above, and then staged and measured each one as above. Then, a colleague uninvolved in the study re-labelled all 20 slides and we re-measured each of the 20 larvae a second time; thus, the second measurement on these same larvae was done ‘blind.’ We calculated measurement error using the difference between each of the paired measurements, and used this error calculation to ensure that any reported differences in L–R asymmetry fell outside of the experimentation error range. We also used these data to assess fluctuating asymmetry (FA) as further explained below.

For the double-rudiment experiment, we staged larvae under a Nikon Eclipse Ti microscope, according to our published staging scheme (see tables 1 and 2 in [22]), and we made three-dimensional ‘z-stacks’ (pictures taken at 10 µm steps through the larvae) with a Nikon Digital Sight DS-Fi1 camera. We then conducted measurements on these z-stacks using a three-dimensional measurement macro (calibrated to account for both the *x*–*y* and *z*–*y* plane distances covered) using the ImageJ software Fiji. We measured left and right postoral (PO), posterodorsal (PD) and anterolateral (ALA) arm lengths for each larva.

**Table 1.** Directional asymmetry in purple urchin larvae by age. Statistics corresponding to the data shown in figures 2*a*–*d* and 4*a*. The ‘regression analysis’ columns show the results of our test to determine if DA changes as a function of age (days PF) using a linear regression. Italicized rows and asterisks in the *p-*value columns denote all cases where *α* was less than 0.05. Abbreviations as in figures 1 and 2. On day 15, these larvae had only six arms; hence the ‘n.a.’ for the preoral (PRO) arms on that day. s.d. = one standard deviation.

					one-sample *t*-test		regression analysis		
arms	age (days PF)	mean ln(*R*/*L*)	s.d.	*n* (larvae)	*t*	*p*-values	*R*^2^	*F*	*p*-values
PO	15	0.030	0.025	9	1.20	0.26	0.01	0.84	0.36
	*25*	*0.050*	*0.021*	*10*	*2.45*	0.037*			
	33	0.042	0.021	30	2.00	0.06			
	*39*	*0.075*	*0.020*	*15*	*3.79*	0.002*			
ALA	15	−0.012	0.026	9	−0.46	0.66	*0.07*	*4.55*	0.04*
	25	0.038	0.035	10	1.09	0.30			
	*33*	*0.191*	*0.037*	*30*	*5.15*	<0.001*			
	*39*	*0.072*	*0.029*	*15*	*2.49*	0.026*			
PD	15	−0.012	0.074	9	−0.16	0.88	*0.09*	*5.77*	0.02*
	25	−0.005	0.024	10	−0.22	0.83			
	*33*	*0.052*	*0.022*	*30*	*2.32*	0.028*			
	*39*	*0.105*	*0.024*	*15*	*4.31*	0.001*			
PRO	15	n.a.	n.a.	n.a.	n.a.	n.a.	0.00	0.24	0.62
	25	0.008	0.021	10	0.40	0.70			
	33	0.050	0.034	30	1.49	0.15			
	39	−0.065	0.055	15	−1.19	0.26			
total (sum of all arms)	15	0.005	0.028	9	0.20	0.85	*0.07*	*4.76*	0.033*
	25	0.025	0.017	10	1.42	0.19			
	*33*	*0.077*	*0.016*	*30*	*4.73*	<0.001*			
	*39*	*0.056*	*0.014*	*15*	*3.99*	0.001*

**Table 2.** Directional asymmetry in purple urchin larvae by stage. Statistics corresponding to the data shown in figures 3*a*–*d* and 4*b*. The ‘regression analysis’ columns show the results of our test to determine if DA changes as a function of stage bin using a linear regression. Italicized rows and asterisks in the *p-*value columns denote all cases where *α* was less than 0.05. Abbreviations as in figure 1. Stage bins as in figure 3.

					one-sample *t*-test		regression analysis		
arms	stage bin	mean ln(*R*/*L*)	s.d.	*n* (larvae)	*t*	*p*-values	*R*^2^	*F*	*p*-values
PO	A	0.031	0.019	14	1.62	0.13	0.00	0.29	0.59
	B	0.018	0.037	7	0.49	0.64			
	C	0.119	0.056	7	2.15	0.075			
	D	0.054	0.030	13	1.82	0.094			
	E	0.025	0.027	8	0.93	0.38			
	*F*	*0.057*	*0.020*	*15*	*2.88*	0.012*			
ALA	A	0.002	0.022	14	0.11	0.91	0.05	2.90	0.09
	B	0.042	0.078	7	0.54	0.61			
	*C*	*0.222*	*0.070*	*7*	*3.17*	0.019*			
	*D*	*0.207*	*0.051*	*13*	*4.06*	0.002*			
	E	0.151	0.081	8	1.87	0.104			
	*F*	*0.087*	*0.029*	*15*	*3.00*	0.010*			
PD	A	−0.008	0.048	14	−0.17	0.87	*0.09*	*5.91*	0.02*
	B	−0.006	0.053	7	−0.11	0.91			
	C	0.052	0.033	7	1.56	0.17			
	D	0.064	0.030	13	2.10	0.06			
	E	0.060	0.059	8	1.01	0.34			
	*F*	*0.097*	*0.019*	*15*	*5.00*	<0.001*			
PRO	A	0.013	0.008	14	1.69	0.12	0.04	2.70	0.11
	B	0.046	0.096	7	0.49	0.65			
	C	0.096	0.066	7	1.44	0.20			
	D	0.044	0.033	13	1.33	0.21			
	E	0.015	0.055	8	0.28	0.79			
	F	−0.085	0.056	15	−1.51	0.15			
total (sum of all arms)	A	0.012	0.020	14	0.62	0.55	0.03	1.88	0.18
	B	0.021	0.020	7	1.03	0.35			
	*C*	*0.119*	*0.032*	*7*	*3.69*	0.010*			
	*D*	*0.086*	*0.023*	*13*	*3.69*	0.003*			
	E	0.058	0.034	8	1.70	0.13			
	*F*	*0.048*	*0.014*	*15*	*3.33*	*0.005*			

**Figure 1.**
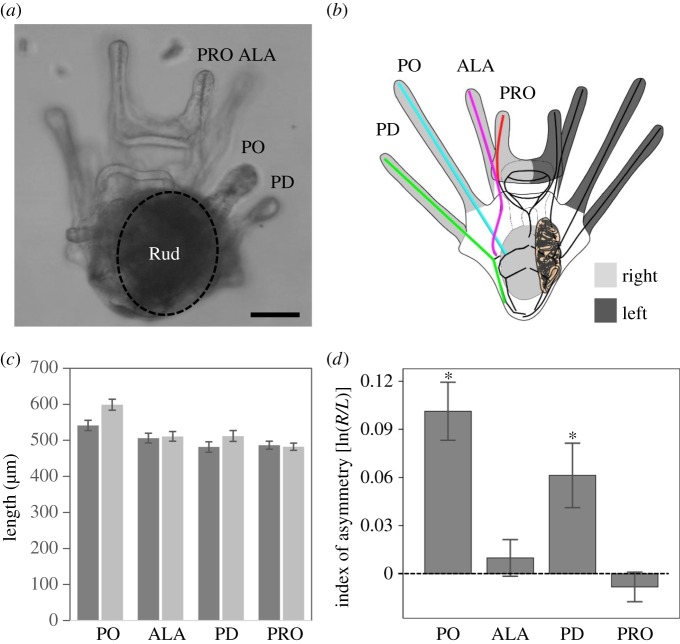
Larvae of the sand dollar *D. excentricus* show directional asymmetry in postoral arms. (*a*) Representative *D. excentricus* larva at 9 days postfertilization, with four pairs of larval arms. Note that the left side of the larva is the side of the well-developed juvenile rudiment (dark region labelled ‘Rud’ in this image); as this is a ventral view, the ‘left’ side of the larvae is seen here on the right side of the image, and vice-versa; scale bar: 100 µm. (*b*) Schematic of a sand dollar pluteus larva with four pairs of larval arms, with the coloured lines indicating the measurements taken for this study, oriented as in (*a*). (*c*) Mean larval arm length in µm for all four larval arms at day 9; *n* = 20 larvae. Lighter bars: right arms; darker bars: left arms. (*d*) Mean index of asymmetry [ln(*R*/*L*)] for all arm pairs; positive values indicate right-based asymmetry (i.e. longer arms on the right side); negative values indicate left-biased asymmetry. PO: postoral arms; PD: posterodorsal arms; ALA: anterolateral arms; PRO: preoral arms. Asterisks in (*d*) indicate significant differences (*p* < 0.05) between left and right side, and therefore directional asymmetry (DA). Error bars are one standard error of the mean.

**Figure 2.**
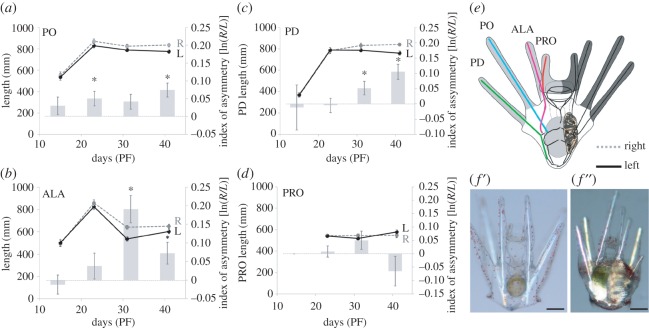
Directional asymmetry in purple urchin (*Str. purpuratus*) larvae as a function of age. We analysed left–right asymmetry in larval arms as a function of age during late-larval development for the (*a*) postoral, (*b*) anterolateral, (*c*) posterodorsal and (*d*) preoral arm pairs (on day 15, these larvae had only six arms; hence the absence of data for PRO arms on that day). Graphs in (*a*–*d*) have two *y*-axes: the primary (left) axis shows larval arm length in micrometres for all four larval arms, as is seen in the data points connected by dark solid (left arm) and dashed grey (right arm) lines. The secondary (right) axis shows the mean index of asymmetry [ln(*R*/*L*)], as is seen in the grey bars. (*e*) Schematic as in figure 1*b* repeated for convenience. The cross-polarized light micrograph in (*f*') shows a representative early stage larva (day 15, stage bin A; see figure 3), and in (*f*''), a representative late-stage larva (day 39, stage bin F; see figure 3). Note the visible juvenile skeleton (on the left side; pictured in the right side of these ventral views) and clear L–R arm asymmetry in (*f*'') but not (*f*'). Time is in days postfertilization (PF), Scale bars in (*e*,*f*), 150 µm. Abbreviations, asterisks and error bars as in figure 1. See table 1 for statistics.

As indicated in figures 1*b* and 2*e*, the PO and PD rods are relatively straight, so our calculation of these skeletal rod lengths was straightforward. By contrast, the preoral (PRO) and ALA rods are somewhat curved in both species. Our reported PRO and ALA rod lengths represent the linear distance between the landmarks indicated in figures 1*b* and 2*e*, and are thus are our best approximations of these rod lengths using the methods we employed.

### 2.3. Statistical analysis

We conducted all statistical tests using SPSS v. 23. We analysed all morphological comparisons using SPSS MANOVA, linear regression and one-sample *t*-test commands. We tested for DA using the index of asymmetry [ln(*R*) − ln(*L*)], which is equal to ln(*R*/*L*), and mathematically equivalent to

2.1 $$\left\{\frac{\left(R-L\right)}{\left[(R+L)/2\right]}\right\},$$

where *R* and *L* are the lengths of the right and left arm, respectively [31]. For each of our structures measured at each time point, we then calculated the mean [ln(*R*/*L*)] and used a two-tailed one-sample *t-*test to determine if this value was significantly different from zero; a positive value indicates right-biased DA, a negative value indicates left-biased DA. We used *q*–*q* and *p*–*p* plots to test for normality throughout. We further tested for age and stage effects on asymmetry using linear regression analysis. We tested for an effect of food on asymmetry with a MANOVA using food level as a factor. We tested for FA using methods outlined by Palmer & Strobeck [31]. In all cases, we considered results ‘significant’ if *α* was less than 0.05. We report all results as ±1 standard error of the mean (s.e.) unless stated otherwise.

## 3. Results

We tested whether echinopluteus larval arms were directionally asymmetrical during mid- to late-larval development, and whether any detected asymmetry changed as a function of developmental age and stage.

### 3.1. Larvae of the sand dollar *D. excentricus* showed directional asymmetry in larval arm growth

We measured all eight larval arms of *D. excentricus* 9 days PF at approximately 20°C; we analysed differences in arm lengths between the left and right side of the larva (figure 1) using the index of asymmetry [ln(*R*/*L*)]. We found that the PO and PD arms of *D. excentricus* larvae were significantly shorter on the side of the juvenile rudiment (left side) compared with the right side (PO: *t* = 5.59, *p* < 0.001; PD: *t* = 3.05, *p* < 0.01; *n* = 20). We did not find differences in arm length between the left and right side of *D. excentricus* larvae for the other two pairs of arms (anterolateral (ALA: *t* = 0.86, *p* = 0.4, *n* = 20); and PRO (PRO: *t* = −0.89, *p* = 0.4, *n* = 20)). We also examined the index of asymmetry [ln(*R*/*L*)] for the sum of all four arm lengths (PO + PD + ALA + PRO) on each side as an indication of the overall asymmetry of the larva, and found that these larvae were indeed significantly asymmetric overall, with shorter total arm length on the left (rudiment) side [ln(*R*/*L*) = 0.04 ± 0.01 (s.e.), *t* = 5.40, *p* < 0.001, *n* = 20].

Note also that we set up fertilizations from two different females in this experiment, and reared their offspring and analysed them separately, to ensure that our results were not an aberration associated with a maternal effect or a given genotype. We did not find a significant interaction between cross and the index of asymmetry for any arm pairs (PO: *F*_1,19_ = 0.71, *p* = 0.41; ALA: *F*_1,19_ = 0.08, *p* = 0.78; PD: *F*_1,19_ = 1.90, *p* = 0.19; PRO: *F*_1,19_ = 0.14, *p* = 0.72), providing no evidence that the asymmetry patterns were different between the two crosses.

### 3.2. Larvae of the purple sea urchin *Str. purpuratus* showed directional asymmetry in larval arm growth that changes through ontogeny

**Figure 3.**
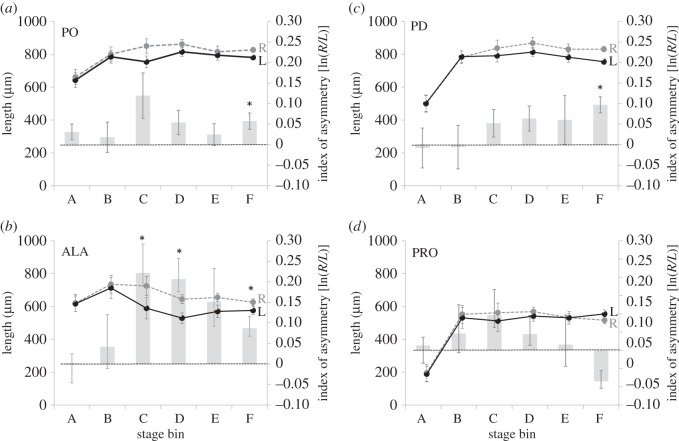
Directional asymmetry in purple urchin larvae as a function of juvenile rudiment stage. We analysed left–right asymmetry in larval arms as a function of juvenile rudiment stage for the (*a*) postoral, (*b*) anterolateral, (*c*) posterodorsal and (*d*) preoral arm pairs. We binned juvenile rudiment stages after [22] as follows: bin A, skeletogenic stage 0; bin B, skeletogenic stages 1–2; bin C, skeletogenic stages 3–4; bin D, skeletogenic stages 5–6; bin E, skeletogenic stages 7–8; bin F, skeletogenic stages 9–10. Double axes and line colours as in figure 2. Abbreviations, asterisks and error bars as in figure 1. See table 2 for statistics.

To determine if the late-stage DAs we observed in sand dollar larvae are a more widespread feature among echinoids, and to examine whether such an asymmetry progresses during ontogeny, we examined DA in the purple sea urchin *Str. purpuratus* at four time points PF. We then analysed arm-length morphometrics as a function of age and binned stage (see figure 3 legend for details).

Based upon the sand dollar data and our preliminary observations on *Str. purpuratus* larvae, we expected DA to first become manifest in later development, after the juvenile rudiment begins to form. Therefore, our earliest measurement date was on day 15, right around the time when we first observed rudiment invagination (figure 2*d*). We made additional measurements during the growth of the juvenile rudiment on days 25, 33 and finally on day 39 (figure 2*e*), at which point the larvae were at or near competence to transform to the juvenile stage (not shown).

DA as a function of age is shown in figure 2 and table 1. Overall, we detected DA starting on day 33 in ALA arms, and in three of the four arm pairs (PO, PD and ALA) on day 39; in each case, the arms were significantly shorter on the left side. To see if DA in each of these arm pairs varied with age, we examined linear regressions for the indices of asymmetry for each of the four sets of arms from day 15 to 39: both the ALA and PD arms showed increasing DA with age (table 1).

To more clearly examine if DA in purple urchin larvae is related to the growth of the rudiment, we sorted all of the larvae (regardless of age) into one of six stage bins (A–F) defined by the growth of skeletal structures in the rudiment (see the legend to figure 3, for details on how stage bins A–F correspond to the rudiment staging scheme in [22]). As seen in figure 3 and table 2, we detected DA in ALA arms in stage bins C, D and F, and in PO and PD arms only in stage bin F (the most advanced larvae in our dataset). As with the age data, we examined linear regressions for the indices of asymmetry for each of the four sets of arms to see if DA in each of these arms pairs varied with stage. In this case, only the PD arms showed increasing DA with stage (table 2).

**Figure 4.**
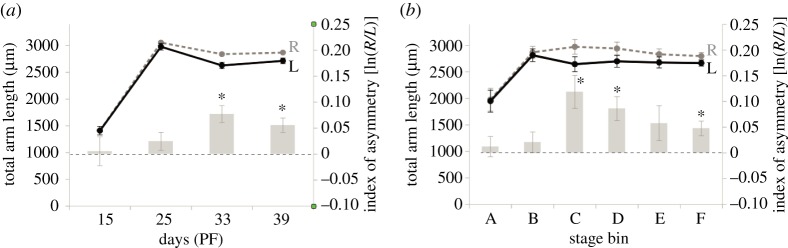
Directional asymmetry in total arm length of purple urchin larvae as a function of age and juvenile rudiment stage. We summed the length of the four pairs of arms on the right and left sides, and compared them by age (*a*) and juvenile rudiment stage (*b*). Note the lack of a monotonic increase in asymmetry as development proceeds, especially in (*b*). Double axes and line colours as in figure 2. Abbreviations, asterisks and error bars as in figure 1. Stage bins as in figure 3. See tables 1 and 2 for statistics.

As different arms show different levels of DA (figures 2 and 3; tables 1 and 2), we analysed the index of asymmetry for the sum of all four arms by both age (figure 4*a*, table 1) and stage (figure 4*b*, table 2). With respect to age (figure 4*a*, table 1), we detected evidence for DA in total arm length on days 33 and 39. A regression analysis showed a positive relationship between index of asymmetry for total arm length and age (table 1).

With respect to stage (figure 4*b* table 2), we detected evidence for DA in total arm length in stage bins C, D and F. But in this case a regression analysis showed no overall relationship between index of DA for total arm length and stage (table 2). As can be seen in figure 4*b*, DA does not monotonically increase with stage; instead the stage of peak DA for total arm length is stage bin C, which is the only stage at which our ANOVA, after Bonferroni correction, detected a significantly higher DA than in any other stage (stage bin C versus stage bin A: *p* = 0.05).

Although it is not the focus of this study, we undertook an analysis of FA in comparison with measurement error. We performed this analysis on a separate cohort of larvae 22 days after fertilization (all of which were in stage bin A; see figure 3 legend). Using a mixed model ANOVA as described by Palmer & Strobeck [31], we found no evidence for DA in PO, ALA or PD arm lengths (electronic supplementary material, table S1; note that these larvae only had six arms at the stage that we examined them, thus we do not have PRO data here). As these larvae were in stage bin A, we did not expect to detect DA in these larvae (cf. figure 3 and table 2). Nevertheless, FA was significantly larger than the measurement error for all arm lengths among these larvae (electronic supplementary material, table S1). Although not entirely comparable, as these larvae were a separate cohort from the main experiment outlined above, we note that the scope of our measurement errors for PO, ALA and PD were 4–5× lower than the scope of L–R differences that are seen in figures 2 and 3.

In sum, we see clear evidence for DA in multiple arms through ontogeny, whether viewed by age or stage. Arm length asymmetry in *Str. purpuratus* larvae increased with age and stage in ALA and PD arms, and total arm asymmetry (i.e. asymmetry in the arms as a whole) also increased with age. When analysed by stage, however, the pattern appears more complex: the greatest degree of total arm asymmetry occurred in our stage bin C, which falls at about the midpoint of rudiment development towards metamorphic competence.

### 3.3. Food did not affect larval arm asymmetry in *Str. purpuratus*

One hypothesis that could explain the previously noted directional asymmetries is that there is competition for limited resources or materials between the rudiment and the nearby left arms. If so, we might expect to observe a more dramatic asymmetry in larvae raised in food-limited conditions, where such materials/resources would be in reduced supply.

To test this hypothesis, we reared larvae under a high food ration for 25 days, and then shifted a subset of the larvae into a reduced food ration (25% of the high food ration) for the remainder of larval development. We then analysed changes in arm length as a function of age and stage for the low food- and high food-reared larvae.

**Figure 5.**
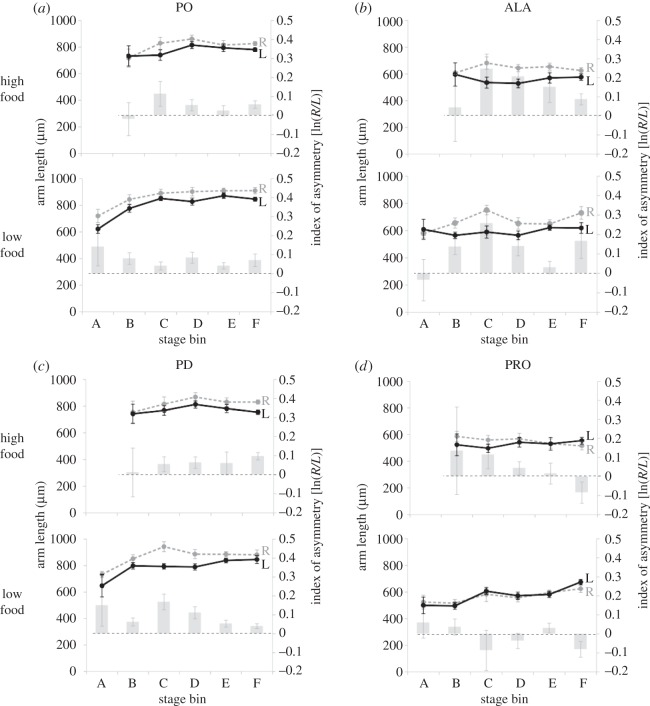
Reduced larval food does not alter directional asymmetries in arm length across stages. (*a*–*d*) We detected asymmetry in arm lengths with stage under both high food (HF; upper graphs in each panel) and low food (LF; lower graphs) conditions in purple urchins, with no detectable differences in any arm pair between high and low food (see the text). Double axes and line colours as in figure 2. Abbreviations, asterisks and error bars as in figure 1. Stage bins as in figure 3. Numbers of larvae at each stage are as follows. Stage bin A: HF, *n* = 0; LF, *n* = 4. Stage bin B: HF, *n* = 3; LF, *n* = 18. Stage bin C: HF, *n* = 6; LF, *n* = 9. Stage bin D: HF, *n* = 13; LF, *n* = 17. Stage bin E: HF, *n* = 8; LF, *n* = 18. Stage bin F: HF, *n* = 15; LF, *n* = 8. Note that there were no HF larvae in stage bin A, presumably due to their more rapid development than in the corresponding low food larval cohort.

Plasticity in larval arm growth overall as a function of food level has been well demonstrated in *Str. purpuratus* and numerous other echinoids (reviewed in [23]): in general, larvae under low food conditions have longer arms relative to growth of juvenile structures. We observed clear arm length plasticity for all arm pairs as a function of binned stage (figure 5; PO: *F*_1,89_ = 21.19, *p* < 0.01; ALA: *F*_1,89_ = 3.85, *p* = 0.05; PD: *F*_1,89_ = 11.97, *p* < 0.01; PRO: *F*_1,89_ = 5.62, *p* = 0.02) as expected.

We then tested whether food environment would impact the extent of arm-length DA in *Str. purpuratus* larvae (figure 5). Statistically, such a food effect on asymmetry would manifest as a significant interaction between food treatment (high or low) and the index of DA [ln(*R*/*L*)]. Note that we only analysed our data by stage, as low and high food larvae developed on quite different trajectories towards metamorphic competence (see Material and methods, above), and it seemed most sensible to normalize by juvenile rudiment stage. We did not find evidence for a statistically significant interaction between food treatment and DA for any larval arms by stage (figure 5; PO: *F*_1,119_ = 0.58, *p* = 0.45; ALA: *F*_1,119_ = 0.01, *p* = 0.98; PD: *F*_1,119_ = 1.44, *p* = 0.23; PRO: *F*_1,119_ = 3.32, *p* = 0.07). DA in the high and low food-treated larvae for total arm length by stage can be found in the electronic supplementary material, figure S1.

As a side note, we observed that arm lengths under high food conditions (figures 2–5) plateaued or in some cases decreased slightly as ontogeny proceeded, whereas under low food conditions, arm lengths continued to increase over the course of the experiment (figure 5). This difference was particularly clear when examining the total (sum) of the lengths of all arms combined (electronic supplementary material, figure S1). We interpret this apparent cessation of arm growth in the context of phenotypic plasticity to differing food levels: under high food, larvae at late stages shifted their investment from arm growth to rudiment growth, as has been seen repeatedly in a variety of echinoids (see e.g. [9,23]).

### 3.4. *Strongylocentrotus purpuratus* larvae with double rudiments do not exhibit right-biased directional asymmetry

**Figure 6.**
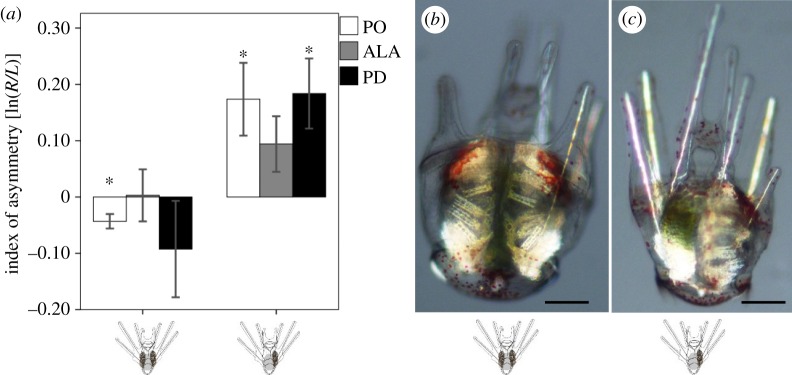
Larval arms of anomalous purple urchin larvae with double rudiments are more symmetrical. We analysed the index of arm-length asymmetry (see figure 1 legend) in naturally occurring larvae with juvenile rudiments on both the left and right sides (double rudiments; (*b*), as compared to their full siblings with single rudiments (*c*). Cartoons along bottom of figure indicate single and double rudiments. (*a*) Whereas larvae with single rudiments (*n* = 9 larvae) in this experiment showed right-biased directional asymmetry (positive values) in postoral (PO) and posterodorsal (PD) arms (asterisks in right half of (*a*)), stage-matched larvae with double rudiments (*n* = 8 larvae) did not show right-biased asymmetry in any of their arm pairs; in fact PO arms in double-rudiment larvae showed left-biased asymmetry (negative values; asterisk in left half of (*a*)). Scale bars in (*b,c*): 150 µm. Abbreviations, asterisks, error bars and orientation of larvae as in figure 1.

We analysed anomalous larvae with naturally occurring double rudiments (i.e. larvae with ‘twin’ juvenile rudiments developing simultaneously on the right and left sides; figure 6*b*) to test the hypothesis that DA in larval arms is functionally linked to the formation of the juvenile rudiment (figures 3 and 4*b*). As expected, our single rudiment larvae (figure 6*c*) showed right-biased DA in both PO and PD arms, with shorter arms on the left side (figure 6*a*; PO: *t* = 2.68, *p* = 0.03; ALA: *t* = 1.90, *p* = 0.09; PD: *t* = 2.95, *p* = 0.02; *n* = 8). By contrast, in our double-rudiment larvae (figure 6*b*), the ALA and PD arms did not show any evidence of DA (figure 6*a*; ALA: *t* = 0.07, *p* = 0.95; PD: *t* = −1.08, *p* = 0.32; *n* = 9). In fact we detected a significant DA in the opposite direction in the PO arms, which had slightly (approx. 4.3%) longer arms on the left side (figure 6*a*; PO: *t* = −3.38, *p* = 0.01, *n* = 9).

## 4. Discussion

Metamorphosis in extant echinoderms involves a transformation from a bilateral larva to a pentameral adult [32,33]. Because the common ancestor of echinoderms and other deuterostomes is hypothesized to have had an adult with bilateral symmetry (reviewed in [34]), this bilateral-to-pentameral shift during echinoderm ontogeny is considered key to understanding the evolution of this unique group [35–37].

In all living echinoderms with indirect development (*sensu* McEdward & Janies [6]), the adult body plan develops in a curious fashion with respect to that of the larva, via an internal DA: the juvenile forms on the left side of the otherwise bilaterally symmetrical larva [18–22]. Here, we show for the first time that in two disparate echinoids, a sea urchin and a sand dollar, a second DA is apparent during late-larval development, when the overall shape of the larva changes from more or less bilaterally symmetric to directionally asymmetric during later stages: the majority of the larval arms, which are supported by calcium carbonate skeletal rods, are substantially shorter on the rudiment side of the larva (up to 25% shorter; see figures 1 and 2).

Although asymmetries in larval arms have been noted previously during normal development in echinoid larvae [38–40], ours is the first demonstration of such a unidirectional asymmetry in multiple arms, resulting in a consistent yet previously undescribed shape change during late pluteus development. As in one other reported case of a DA in advanced pluteus larval arms [39,40], we show evidence here for a link between formation of the rudiment and the arm-length asymmetry, with shorter larval arms specifically on the rudiment (left) side. Our demonstration of this asymmetry in both an irregular and a regular echinoid, as well as our casual observations of such asymmetries in the larvae of several other echinoid taxa which we have reared from several geographically disparate locations (data not shown), suggest to us that this DA in arm length is probably a generic feature of late-stage, echinoid larval development, and as such, it calls out for a functional explanation.

### 4.1. Possible developmental mechanisms underlying larval arm asymmetry

Based on previous studies, Nodal and BMP signalling are the primary factors responsible for the development of the juvenile rudiment on the left side of the sea urchin larva, with Nodal expression on the right inhibiting BMP signalling there, and thus directing BMP-activation of rudiment formation to the left side [24,25,41,42]. Still, the consequences of this asymmetry for development of the larval arms are unclear.

If the BMP–Nodal gene network is likewise involved in our reported arm-length asymmetries, then we would predict that the arm-length asymmetries would coincide with rudiment formation. This is not what we have observed. Indeed, at soft tissue stage iv [22] (stage bin A, day 25 in this experiment) rudiment formation was already well underway and we did not observe any directional asymmetries in arm length, which we first detected at skeletal stage 3–4 [22] (stage bin C, approx. day 31 in this experiment; figures 2–4).

These observations suggest that if the BMP–Nodal gene network is involved in the directional arm asymmetry, then this involvement is likely to be either indirect, or to involve subsequent signalling steps. Still, our data reported here on double rudiments do suggest that there is a mechanistic connection between rudiment asymmetry and the arm-length DA, as also reported by Emlet [40] for the posterolateral arm-length asymmetry in the black sea urchin, *Stomopneustes variolaris* (Echinoidea: Stomopneustidae). Evaluating the nature of this mechanistic connection could involve examining arm asymmetries in the context of reduction-of-function manipulations (e.g. using morpholino oligonucleotides) directed against BMP or Nodal signalling components. Specifically, one could experimentally generate double-rudiment larvae, for example by inhibiting Nodal signalling [24], and test whether such a manipulation results in loss of the DA, as we saw in our naturally occurring double-rudiment larvae. If BMP–Nodal signalling is indeed connected to the arm-length asymmetries, then it is possible that the expression of skeletal elongation genes such as *p58b* and *p16* [43,44], and the skeletogenic gene network to which they belong [45], may integrate with the BMP–Nodal gene network in an as yet unrecognized fashion.

### 4.2. Functional implications of the asymmetry for larval biology

The antagonistic relationship between rudiment growth and arm length in feeding pluteus larvae has been well described in numerous taxa. Specifically, echinoid larvae show adaptive phenotypic plasticity for arm growth relative to growth of the rudiment: under high food conditions, arm length is reduced relative to rudiment growth; under low food, arms grow longer and rudiment growth is delayed [9,23,46–64]. The adaptive nature of this plasticity is indicated by the increased food capture efficiency in larvae with longer arms [9].

Therefore, there is presumably a mechanistic connection between rudiment growth and arm length, and it may be that this same antagonistic connection underlies the late stage arm-length asymmetries reported here and by Collin [38], Yanagisawa [39] and Emlet [40]. But why would such an antagonistic connection only manifest on the left side of the larva during these late stages? Here, we consider two functional hypotheses in turn for our observed directional asymmetries in arm length. According to the *developmental constraint hypothesis*, the asymmetries that we report here result from some type of local developmental constraint or trade-off, in which the rudiment grows at the expense of only the adjacent larval arms. Alternatively, the *swimming stability hypothesis* predicts that this link is driven by selection on a specific, asymmetric larval shape that facilitates stability or some other aspect of performance in the water column.

The *developmental constraint hypothesis* envisions a scenario where some factor utilized in the construction of both the rudiment and the larval arms is in limited supply. The increasing demands in the rapidly growing rudiment for this hypothesized factor leaves less of it available for the growth of nearby larval arms, and the result is shorter larval arms adjacent to the rudiment.

Although our experiments are not sufficient to falsify this hypothesis, our results are not wholly consistent with it. On the one hand, the left larval arm that is most distant from the rudiment—the left PRO arm—shows no evidence for DA in either *D. excentricus* or *Str. purpuratus*, thus offering some support for a local constraint related to the rudiment. Furthermore, the constraint hypothesis would predict that the local competition would become more pronounced as the rudiment grows ever larger and more complex as ontogeny proceeds; our developmental time series with *Str. purpuratus* (figure 2 and table 1) is more or less consistent with this prediction. However, when we analysed our larvae grouped by rudiment development stage (figure 3 and table 2), we detected no increase in asymmetry at later stages. Indeed, the stage with the most dramatic asymmetry was stage bin C, about mid-way through rudiment development, where calcification of juvenile structures in the rudiment is at an early stage [22]. Furthermore, under a reduced food scenario, where arms grow longer and rudiment growth is delayed, the constraint hypothesis would seemingly predict an even more dramatic asymmetry than in our well-fed larvae. This is not what we observed; we saw no difference in the observed asymmetry between food treatments.

Therefore, our data provide mixed support for the constraint hypothesis. Still there is some precedence in other developing organisms for such a hypothesized local competition for factors or resources. For example, the wing-reproduction trade-off in monarch butterflies (*Danaus plexippus*) has been proposed to be related to a position-dependent mechanism, where juvenile hormone—produced in the brain—travels in the hemolymph past the wings and through the thorax, ultimately reaching the gonads in the posterior. Lessman & Herman [65] hypothesized that the highly active thorax during long-distance flight acts like a gauntlet, breaking down active juvenile hormone as it passes, leaving lower levels to arrive at the gonad, and therefore leading to reduced gonadal growth during flight. A second proposed example is in horned beetles (*Onthophagus spp.*) and other insects, where a factor such as insulin may be in limited supply, and could account for the apparent trade-off in the sizes of adjacent structures ([66–68]; but see [69]).

In the urchin pluteus example, what could such a factor be? One appealing possibility relates to the availability of calcium, which is used to construct both the skeletal rods that support the larval arms and the diverse juvenile skeletal elements forming in the rudiment at late-larval stages; these processes may thus be in direct, local competition. A simple experiment that would test for calcium limitation would be to add additional calcium to the seawater in which the larvae are grown—if calcium within the larvae at these late stages is normally in limited supply, then the asymmetry could be attenuated or disappear if excess calcium is provided. Alternatively, one could block calcium transport into the tissues of the rudiment through morpholino microinjection [70] or treatment with chemical inhibitors, and test whether such treatments result in more symmetrical larvae than in controls. Indirect evidence for this calcium limitation hypothesis follows from the findings of Byrne *et al*. [71], who reported that *Heliocidaris tuberculata* (Echinoidea: Echinometridae) larvae reared at low pH showed notable asymmetries (presumably FA) at early larval stages.

A second intriguing possibility is that the local rudiment–arm-length antagonism is regulated by thyroid hormone (TH) signalling. Our previous studies [59] demonstrated that TH treatment results in a phenotype similar to that previously described for low food treatments, where juvenile structures grow faster, and larval arm growth is suppressed. Experiments with TH synthesis inhibitors [33,72–74] indicate that feeding larvae have the capacity to produce TH internally, and our unpublished immuno-labelling experiments indicate that the source for TH may be structures within the rudiment. Therefore, if the rudiment is indeed the source for TH in plutei, and if TH levels correlate negatively with arm growth, then one might expect arms near to the rudiment to be shorter than ones more distant: this is precisely what we have observed here. A simple test of this scenario would be to provide excess TH exogenously and see if the observed asymmetries disappear.

Finally, additional support for biased L–R allocation of materials in pluteus larvae comes from some intriguing observations on sea star bipinnaria larvae, whose feeding larvae are considered homologous to echinoid plutei [75]. Circulation of fluid in the blastocoel cavity of bipinnaria larvae has been described as largely unidirectional: from the stomach, along the left side of the larva, and then around the mouth to the right side of the larva ([76]; Jaeckle, personal communication). Furthermore, coelomic fluid flow out of the left hydrocoel via the pore canal and hydropore to the exterior of the larva [77] would tend to draw blastocoelar fluid towards the left side [78], with the growing rudiment in later stage larvae thus being a possible sink for blastocoelar substances. This biased directional flow could therefore represent a mechanistic basis for uneven distribution of blastocoelar substances, leading to the asymmetries that we report here.

The *swimming stability hypothesis* proposes that the asymmetry in larval arms would provide a selective advantage to larvae, whereby asymmetrical larvae would, for example, sink more slowly (and thus be retained in the water column more efficiently) than symmetrical larvae. Such an asymmetry would be predictably directional due to the substantial asymmetry in ballast provided by the rapidly growing and calcifying rudiment, predominantly on the left side of the larva.

Indirect support for this hypothesis comes from several examples in the literature. First, Collin [38] detected FA in early, pre-rudiment larval stages of the sand dollar *D. excentricus,* and documented the first indication of a subtle DA in a single arm (PD) during mid- to late-larval development. FA is widely viewed as a measure of developmental stability and perturbations to the developmental process, and can have both genetic and environmental causes [79]. Indeed, several studies have demonstrated or suggested an increase in FA or other asymmetries when sea urchin larvae or adults are exposed to toxins (e.g. [80,81]). Nevertheless, the apparent continuity between FA in larval arms earlier in normal ontogeny [38], and then increasing DA later ([38]; our data reported here) may indicate that the asymmetries themselves may be functional throughout normal larval development. In this case, the forming rudiment in late stages might impose additional constraints that could lead to predictably shorter arms on the left.

Chan [82] reviewed a number of studies on pluteus larval morphology as it relates to stability and swimming, under different flow regimes and through ontogeny. The basic pattern that Chan reports is a slight tilt in the orientation of the larval body in flow increases the chances that a larva can maintain upward swimming (and thus presumably stay in surface waters), rather than being carried downward. In an unpublished study, Miyashita (personal communication) discovered that modelled, asymmetrical *D. excentricus* larvae at the four-arm stage (pre-rudiment growth) are able to maintain upward swimming more effectively than symmetrical ones.

The models that Chan and Miyashita employed were based upon those developed by Clay & Grunbaum [10], again using the sand dollar *D. excentricus*, but focusing only on early (4 arm) larval stages before the development of the rudiment. One important set of parameters in this model relates to the centre of gravity, which would clearly change with the addition of an asymmetric, calcified rudiment, as seen in the stages that we examined (though *D. excentricus* larvae become surprisingly more buoyant as ontogeny proceeds; [83]). Furthermore, drag on larvae increases with both arm length and arm number [15], which would be predicted to impact the stability of larvae in different flow regimes [8].

Taken together, the studies to date suggest that larval shape, orientation and asymmetries all can contribute to position in the water column, which can have important consequences for dispersal or near shore retention, prey encounter and predator avoidance throughout larval development, and contacting the substrate in late-stage larvae preparing to settle to the benthos. Nevertheless, it is difficult to extrapolate from the previous modelling studies on much simpler larval morphologies to those in fully formed, eight-arm larvae with an asymmetrically growing and calcifying rudiment in realistic flow conditions.

Our experiments reported here do not directly address the swimming stability hypothesis. However, one intriguing observation is that the three arm pairs that protrude the furthest from the larval midline—the PO, PD and ALA arms—are the three pairs that showed clear directional asymmetries in one or both species. By contrast, the arm pair that runs closest to the midline—the PRO arms—showed no signs of DA in either species at any age or stage that we examined. Likewise, the observations by Yanagisawa [39] and Emlet [40] of a dramatic asymmetry in late-stage larvae of the sea urchin *Sto. variolaris* was specifically in a unique pair of arms that project off the posterior end of those larvae, the posterolateral arms. All of these observations suggest that asymmetric arm growth is not simply a generic feature of late-stage pluteus larval arm growth common to all arms, and may thus point towards a functional explanation such as envisioned by the swimming stability hypothesis.

To adequately test this hypothesis experimentally, one would need to examine larvae under realistic flow conditions, and see if the degree of asymmetry in late-stage larvae is related to their position in the water column. In addition, one could develop more complex models of the pluteus larval form that would extend from the Clay & Grunbaum [10] model, but include all four arm pairs and a growing and calcifying, asymmetrical rudiment. The swimming stability hypothesis would predict that arm asymmetries at these later stages would have clear consequences for position in the water column and/or swimming ability.

Finally, we note that the developmental constraint and swimming stability hypotheses are not the only two possible explanations for our observed asymmetries (and these themselves are not necessarily mutually exclusive). For example, in recent years it has become clear that asexual larval cloning is widespread in echinoids (which again, has been particularly well documented in *D. excentricus*), and one method of such asexual cloning in larval sea stars [84,85]—as yet undocumented in larval echinoids (but see [28])—is budding of the arm tips. It is possible that such budding occurs preferentially on the left side of late-stage echinoid larvae, which would lead to a DA pattern such as we observed. Furthermore, Emlet [40] suggested that the asymmetry in posterolateral arms in *Sto. variolaris* may be an adaptation for more effective settlement to the benthos: a long left posterolateral arm could interfere with substrate contact. A similar mechanical interference scenario might promote the evolution of shorter arms in late stages in other urchins as well, as we have observed here.

## 5. Conclusion

We here identify and characterize the ontogeny of a DA in the shape of echinoid pluteus larvae that is visible at late stages, alongside the well-known internal DA of the growing juvenile rudiment. Our data from two disparate echinoids, separated by approximately 250 million years of evolution, suggests that this consistent, previously undescribed asymmetry in multiple arms, and hence in overall larval shape, may in fact be a common feature of late-stage echinopluteus ontogeny. By examining rare, anomalous plutei with juvenile rudiments on both the left and the right side, we show that the asymmetry is mechanistically and/or functionally connected to rudiment development. We explore several hypotheses to account for this asymmetry, focusing on two main hypotheses: that the asymmetry aids in swimming stability in the water column or that it is a result of a developmental constraint on material deposition in arms versus the rudiment.

One feature of echinoids that makes them such a valuable taxon for comparative studies is their great morphological diversity in the context of a relatively robust understanding of their phylogeny. As with adult features, sea urchin larvae also show remarkable diversity: for example, in arm number, their lengths relative to the body and their position [86]. Furthermore, there are many independently evolved instances of loss of larval feeding, accompanied by partial to complete loss of these larval arms [87]. And finally, functional and anatomical studies indicate that the similar larval morphology in the brittle stars (class Ophiuroidea) represents a completely independent evolutionary acquisition of the pluteus form [12,13]. Such diversity in form, with independent evolutionary events and an easily quantifiable morphology, provides ample material for detailed comparative investigations into this DA: a tractable aspect of functional morphology that can be studied in the context of the multiple ecological requirements facing feeding and dispersing larvae in the ocean.

## Supplementary Material

Figure S1: Reduced larval food does not alter directional asymmetries in total arm length across stages. We detected asymmetry in total arm length with stage under both high food (upper graph) and low food (lower graphs) conditions in purple urchins, with no detectable differences in any arm pair between high and low food (see Results). Double axes and line colors as in Fig. 2. Abbreviations and error bars as in Fig. 1. Stage bins as in Fig. 3. Numbers of larvae as in Fig. 5. Note that there were no high food larvae in stage bin A, presumably due to their more rapid development than in the corresponding low food larval cohort.

## Data accessibility

All relevant data are included in this article. We submitted all raw data used for the analysis presented in this study to Dryad (http://dx.doi.org/10.5061/dryad.4nj8n) in accordance with RSOS policy.
