# Supplementary material for: A newly identified left–right asymmetry in larval sea urchins
Source: R Soc Open Sci. 2016 Aug 31;3(8):160139. doi: 10.1098/rsos.160139 (PMC5108941; doi:10.1098/rsos.160139)
Supplement: Figure S1: Reduced larval food does not alter directional asymmetries in total arm length across stages. We detected asymmetry in total arm length with stage under both high food (upper graph) and low food (lower graphs) conditions in purple urchins, with no detectable differences in any arm pair be [file rsos160139supp1.doc]

**Table S1: Analysis of fluctuating asymmetry (FA) and directional asymmetry (DA) in comparison to measurement error in purple urchin larvae.** Note that we conducted this measurement error analysis on a separate batch of larvae from those used in the main experiment, and that these larvae were all in Stage Bin A (see Figure 3 legend), and had not yet formed preoral (PRO) arms. Bold rows and asterisks in the FA column denotes all cases where α was < 0.05.

| Arms | Factor | MeanSquare | df | FA | DA |
| --- | --- | --- | --- | --- | --- |
| PO | Side | 79.14 | 1 | **F_18,38_=6.32**  ***p*<0.01*** | F_1,18_=0.06  p=0.80 |
|  | Individuals | 8327.54 | 18 |  |  |
|  | Side x Individual | 1247.35 | 18 |  |  |
|  | Error | 197.415 | 38 |  |  |
| ALA | Side | 475.603 | 1 | **F_18,38_=3.87**  ***p*<0.01*** | F_1,18_=0.59  p=0.45 |
|  | Individuals | 6949.59 | 18 |  |  |
|  | Side x Individual | 810.45 | 18 |  |  |
|  | Error | 209.38 | 38 |  |  |
| PD | Side | 3763.49 | 1 | **F_18,38_=19.31**  ***p*<0.01*** | F_1,18_=0.98  p=0.33 |
|  | Individuals | 13079.48 | 18 |  |  |
|  | Side x Individual | 3834.53 | 18 |  |  |
|  | Error | 198.57 | 38 |  |  |


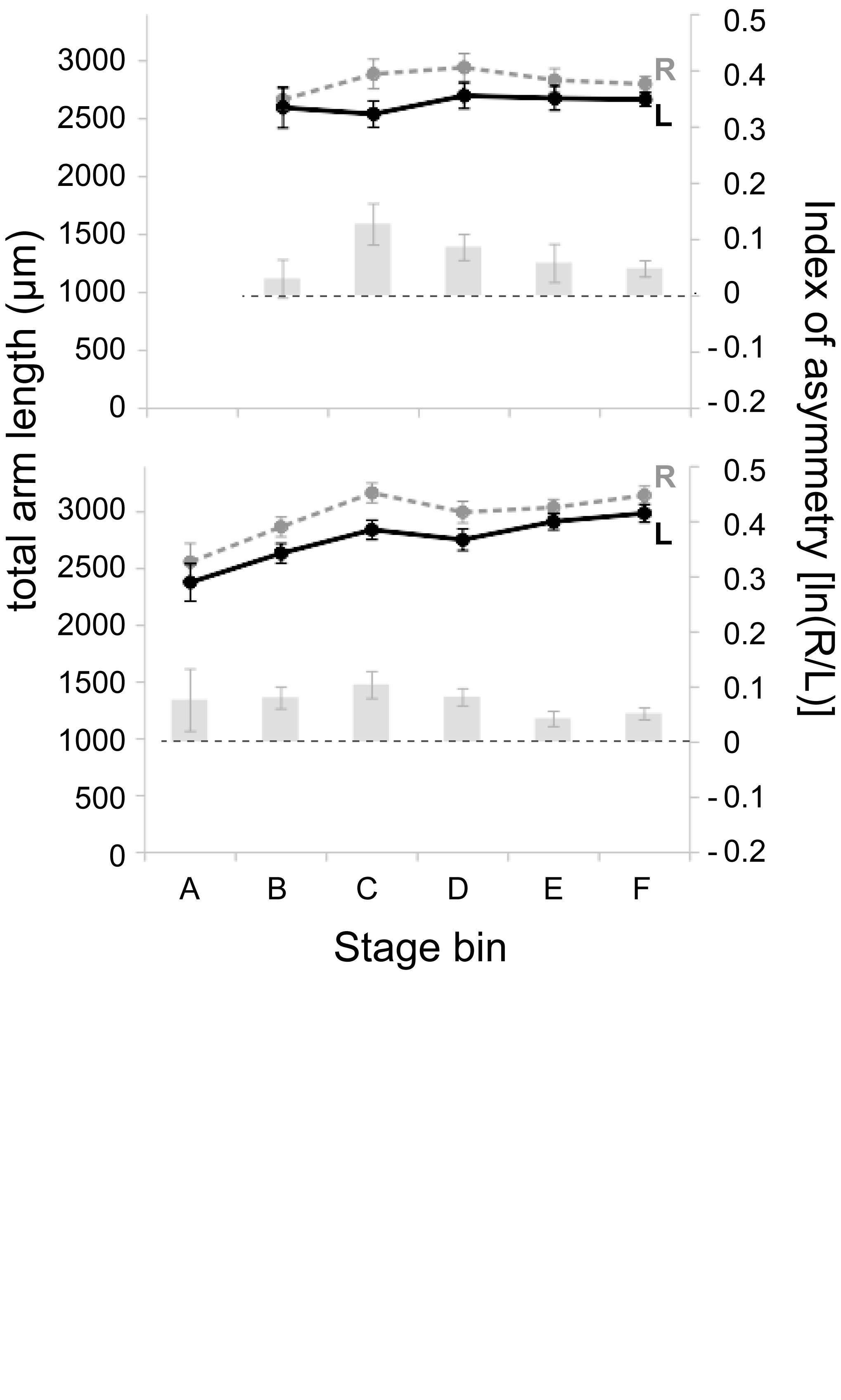


**Figure S1: Reduced larval food does not alter directional asymmetries in total arm length across stages.** We detected asymmetry in total arm length with stage under both high food (upper graph) and low food (lower graphs) conditions in purple urchins, with no detectable differences in any arm pair between high and low food (see Results). Double axes and line colors as in Fig. 2. Abbreviations and error bars as in Fig. 1. Stage bins as in Fig. 3. Numbers of larvae as in Fig. 5. Note that there were no high food larvae in stage bin A, presumably due to their more rapid development than in the corresponding low food larval cohort.
